# Associations Between Alcohol Consumption Patterns and Dyslipidemia Among Chinese Adults Aged 18 and Above: China Nutrition and Health Surveillance (2015–2017)

**DOI:** 10.3390/nu17193112

**Published:** 2025-09-30

**Authors:** Xiaoli Xu, Shujuan Li, Huijun Wang, Qiya Guo, Hongyun Fang, Lahong Ju, Xue Cheng, Weiyi Gong, Xiaoqi Wei, Wenwen Du, Jiguo Zhang, Aidong Liu

**Affiliations:** 1 National Institute for Nutrition and Health, Chinese Center for Disease Control and Prevention, Beijing 100050, China; xuxl@ninh.chinacdc.cn (X.X.);; 2 Key Laboratory of Public Nutrition and Health, National Health Commission of the Peoples’ Republic of China, Beijing 100050, China

**Keywords:** alcohol consumption, hypercholesterolemia, hypertriglyceridemia, low high-density lipoprotein cholesterolemia (low HDL-C), high low-density lipoprotein cholesterolemia (high LDL-C), China

## Abstract

**Background/Objectives:** Alcohol consumption can increase the risk of dyslipidemia, thereby elevating the risk of cardiovascular diseases. However, the relationship between alcohol consumption patterns and dyslipidemia remains controversial. Based on large-scale cross-sectional data from the Chinese population, this study aims to investigate the correlations between various alcohol consumption behaviors and dyslipidemia among adult residents in China. **Methods**: Our analysis utilized data from the 2015–2017 China Nutrition and Health Surveillance project, which provides a large, nationally representative sample (*N* = 52,471). We employed a binary logistic regression model specifically designed for complex sampling frameworks. This model was utilized to assess the relationship between various alcohol consumption behaviors (including daily alcohol intake levels and drinking frequency) and the incidence of hypercholesterolemia, hypertriglyceridemia, low levels of high-density lipoprotein cholesterol (low HDL-C), and elevated levels of low-density lipoprotein cholesterol (high LDL-C). Drinking behaviors were classified into three distinct categories for analysis: China classification (never, moderate, excessive), WHO classification (never, low-risk, medium-risk, high-risk), and drinking frequency (never, <1, 1–3, 4–6, ≥7 times/week). **Results**: Compared with never drinkers, the risk of hypercholesterolemia was significantly higher in men who were excessive drinkers (aOR = 1.39, 95%CI: 1.24–1.57), medium-risk drinkers (aOR = 1.24, 95%CI 1.01–1.53), high-risk drinkers (aOR = 1.67, 95%CI: 1.4–1.95), and those who drank more than once a week (aOR range: 1.27–1.65), and there was no such association in women (*p* > 0.05). Compared with never drinkers, the risk of hypertriglyceridemia was higher in male drinkers with excessive drinking (aOR = 1.35, 95%CI: 1.24–1.47), medium-risk drinking (aOR = 1.29, 95%: 1.11–1.50), high-risk drinking (aOR = 1.52, 95%CI: 1.3–1.71), and a drinking frequency more than 1 time/week (aOR range: 1.22–1.38), while in women, it was moderate drinking (aOR = 0.85, 95%CI 0.77–0.94), low-risk drinking (aOR = 0.86, 95%CI 0.78–0.94), and a drinking frequency of more than once a week (aOR = 0.74, 95%CI 0.63–0.87) that reduced the occurrence of hypertriglyceridemia. Compared with non-drinkers, men with any drinking status had a lower risk of low HDL-C (aOR range: 0.38–0.90) and a similar association was also observed in women (aOR range: 0.26–0.84). Compared with never drinkers, male excessive drinkers (aOR = 0.86, 95%CI: 0.77–0.97), medium-risk drinkers (aOR = 0.80, 95%CI:0.65–0.99), high-risk drinkers (aOR = 0.83, 95%CI: 0.70–0.97), and those with a drinking frequency of 1–3 times/week (aOR = 0.89, 95%: 0.79–0.99) had a lower risk of high LDL-C, and there was no such association in women (*p* > 0.05). **Conclusions**: Significant gender differences were observed in the effects of alcohol consumption on lipid profiles. Men who were excessive drinkers, medium-risk drinkers, high-risk drinkers, and those who drank more than once a week had a higher risk of hypercholesterolemia and hypertriglyceridemia, but a lower risk of low HDL-C and high LDL-C. In women, moderate drinking was associated with a reduced risk of hypertriglyceridemia. Any alcohol consumption and drinking frequency more than 1 time/week were associated with a lower risk of low HDL-C in women. No significant association was found between alcohol consumption and hypercholesterolemia or high LDL-C in women.

## 1. Introduction

The World Health Organization (WHO) “Global Status Report on Alcohol (2018)” points out that more than 2.3 billion people (aged > 15 years) worldwide engaged in drinking behavior in 2016. Drinkers consume more than 35 billion liters of pure alcohol per year, equivalent to 33 g of pure alcohol per drinker per day [1]. Alcohol consumption has become a prevalent behavior worldwide that is closely associated with various health issues. In recent years, the relationship between alcohol consumption and metabolic diseases, such as dyslipidemia, has attracted extensive attention, following the change in lifestyles and the expansion of the drinking population. Dyslipidemia, an important risk factor for cardiovascular diseases [2,3,4,5,6], is characterized by elevated total cholesterol (TC) and (or) triglyceride (TG), as well as elevated low-density lipoprotein cholesterol (LDL-C) and reduced high-density lipoprotein cholesterol (HDL-C) [7]. The results from a nationwide survey in China showed that from 2002 to 2018, lipid levels among Chinese residents gradually increased; by 2018, more than one-third of Chinese residents aged 18 and above had dyslipidemia [8]. Lifestyle behaviors such as drinking are modifiable and are often regarded as an entry point for the primary prevention of chronic diseases. The current evidence on the relationship between alcohol consumption and dyslipidemia remains inconsistent. Some studies suggest that moderate alcohol consumption may have a protective effect on lipid profiles, such as increasing HDL-C levels, while others indicate that alcohol intake may elevate TC and TG levels, thereby exacerbating dyslipidemia. Additionally, the type of alcohol, the frequency of consumption, and individual differences (e.g., genetic background, dietary habits, and lifestyle) may also lead to varying effects on lipid metabolism. Therefore, further exploring the relationship between alcohol consumption and dyslipidemia is of great significance for developing targeted prevention and intervention strategies.

This study provides evidence for the correlation between alcohol consumption and dyslipidemia by analyzing large-scale population data and offers a scientific basis for the prevention and management of cardiovascular diseases.

## 2. Data Sources and Methods

### 2.1. Study Design

The data was from the 2015–2017 China Nutrition and Health Surveillance project [9]. This was a cross-sectional study led by the National Health Commission of the People’s Republic of China. In 2015, the target population for the surveillance was adults ≥ 18 years. A multistage stratified systematic cluster random sampling design was used to select 302 survey sites within 31 provincial-level administrative divisions (PLADs) in mainland China. At least 270 households were selected at each survey site, including households for dietary survey and non-dietary survey households. The sample was nationally representative, as well as representative of urban and rural areas and provinces (excluding Taiwan, Hong Kong, and Macao). Prior to project initiation, the Chinese Center for Disease Control and Prevention conducted four rounds of national-level training workshops on field investigation techniques. Subsequently, provincial-level training was carried out by local CDC centers in each province (autonomous region/municipality). All investigators were required to pass qualification assessments before being permitted to participate in field investigations. The project was launched in June 2015, and field investigations were conducted from August to November at 302 surveillance sites in China after training the surveyors at all levels. Data pertaining to nutrition and health-related indicators were systematically gathered via structured face-to-face interviews, comprehensive medical examinations, detailed laboratory analyses, and thorough dietary assessments. This study protocol was evaluated by the Ethics Committee of the Chinese Center for Disease Control and Prevention (China CDC) (No. 201519-B). All information was collected by trained investigators, and the participants in this survey voluntarily participated and signed an informed consent form.

For this study, we selected the survey data of participants aged ≥ 18 years who completed the household dietary survey for in-depth analysis. The dietary intake data in this study was collected from a food frequency questionnaire (FFQ). Considering that individuals who were aware of hyperlipidemia and the impact of lipid-lowering medications on lipid levels may actively change their dietary behavior, during the data processing, we excluded not only records with missing or abnormal values in sociodemographic, lifestyle, anthropometric, laboratory, alcohol intake, and other dietary data, but also participants who reported having hyperlipidemia and were taking lipid-lowering medications at the time of the survey. Finally, a total of 52,471 adults aged 18 years and above were included in the study.

### 2.2. Data Collection

The 2015–2017 China Nutrition and Health Surveillance (CNHS) project utilized standardized questionnaires, measurement protocols, and laboratory detection methods to systematically collect data on demographic characteristics, behavioral patterns, dietary intake, height, weight, and detailed blood biochemical indicators. Basic personal information, including age, gender, residential area, and educational background, was obtained through face-to-face interviews using a structured questionnaire. Dietary data were collected via a food frequency questionnaire (FFQ) during face-to-face interviews to obtain the consumption data for staple foods, pulses, vegetables, fruits, milk, meat, seafood, eggs, beverages, alcohol consumption, and other food groups over the preceding 12 months. Participants were asked to recall their consumption frequency (recorded as times per day, week, month, or year) and the average consumption size each time. The National Institute for Nutrition and Health, China CDC, developed a food atlas for this survey. It featured foods served in commonly used tableware, indicating the weight of different foods both before and after cooking. Investigators used the food atlas to help participants estimate food intake more accurately.

Height and weight were measured according to standardized techniques and all equipment was calibrated before use. Height was measured using a standardized stadiometer (brand and model: TZG, Jiangyin, China) accurate to 0.1 cm, while body weight was measured using an electronic scale (TANITA, HD-390, Dongguan, China) accurate to 0.1 kg. A total of 4 mL of 10–14 h fasting venous blood was collected and placed in a 5 mL separation gel collection tube for the determination of lipid levels. The instruments and reagents used for blood lipid testing had complete generation licenses, medical device registration certificates or National Food and Drug approval numbers, and business licenses, which were in line with the standards and access scope of the relevant Chinese authorities. Four blood lipid parameters were uniformly measured at the laboratory of the National Institute for Nutrition and Health, China CDC, using a Hitachi 7600 automated biochemical analyzer (Tokyo, Japan). All reagents were procured through official channels. Cholesterol (TC) was measured using the cholesterol oxidase method, with a linear range of 0.10–20.70 mmol/L; triglyceride (TG) was measured using the free glycerol removal method, with a linear range of 0.01–22.60 mmol/L; high-density lipoprotein cholesterol (HDL-C) was measured using a direct assay with the antibody inhibition method, exhibiting a linear range of 0.08–3.88 mmol/L; and low-density lipoprotein cholesterol (LDL-C) was determined by a direct assay employing a selective protection method, with a linear range of 0.10–14.20 mmol/L.

### 2.3. Diagnosis of Dyslipidemia

The diagnosis of dyslipidemia in adults was based on the “Chinese Guidelines for Lipid Management” (2023) [7].

(1) Serum total cholesterol level (TC) ≥ 6.2 mmol/L was diagnosed as hypercholesterolemia;
(2) Serum triglyceride level (TG) ≥ 2.3 mmol/L was diagnosed as hypertriglyceridemia;
(3) High-density lipoprotein cholesterol level (HDL-C) < 1.0 mmol/L was diagnosed as low HDL-C;
(4) Serum low-density lipoprotein cholesterol level (LDL-C) ≥ 4.1 mmol/L was diagnosed as high LDL-C.

### 2.4. Assessment of Alcohol Intake and Grouping

We investigated the drinking situation of six kinds of alcoholic beverages: high-proof liquor, low-proof liquor, beer, rice wine, yellow wine, and wine. The alcohol content of the six beverages is defined as 52%, 38%, 4%, 18%, 18%, 14%, respectively. The daily average alcohol intake was calculated based on the alcohol content of the different types of alcoholic beverages.

We split the daily alcohol intake levels into two types based on the “Dietary Guidelines for Chinese Residents (2022)” [10] and the “ Alcohol Consumption and Related Harm Monitoring Guidelines” published by the WHO [11]: Chinese classification and WHO classification. The Chinese classification of daily alcohol intake was divided into three categories: never consumes alcohol (daily alcohol intake of 0), moderate alcohol consumption (0 g < daily alcohol intake < 15 g), and excessive alcohol consumption (daily alcohol intake ≥ 15 g). The WHO classification of daily alcohol intake was never consumes alcohol (daily alcohol intake of 0), low-risk alcohol consumption (male: 0 g < average daily alcohol intake < 41 g or female: 0 g < average daily alcohol intake < 21 g), medium-risk alcohol consumption (male: 41 g ≤ average daily pure alcohol intake < 61 g or female: 21 g ≤ average daily alcohol intake < 41 g), and high-risk alcohol consumption (male average daily alcohol intake ≥ 61 g, or female average daily alcohol intake ≥ 41 g). The drinking frequency was divided into never, <1 time/week, 1~3 times/week, 4~6 times/week, and drinking every day. Due to the low frequency of alcohol consumption among women, in a study exploring the blood lipid levels of women under different alcohol consumption patterns and the relationship between alcohol consumption frequency and blood lipids, alcohol consumption frequency was divided into never drinking, <1 time/week, and ≥1 time/week.

### 2.5. Variables of Influencing Factors

In studies analyzing the effect of drinking behavior on dyslipidemia, confounding influence variables were selected based on the risk factors and protective factors that have been studied that may affect lipid levels. We considered four basic sociodemographic characteristics: age, region, cultural level, and weight, as well behavioral factors such as smoking status, physical activity status, and dietary intakes.

Age was categorized into five groups: 18–44 years old, 45–59 years old, 60–69 years old, 70–79 years old, and ≥80 years old. Regions were classified as urban and rural based on population size and the NBS definitions of urban or rural. According to the level of education received by the respondents, they were divided into six groups: not receiving formal education from school, primary school, junior high school, high school, technical school, and college or above. Weight status was classified according to the Chinese health industry standard “Adult Weight Standard” (WS/T 428-2013) [12]. Body mass index (BMI) was used to distinguish weight. BMI = weight/height 2 (kg/m^2^), obesity: BMI ≥ 28.0, overweight: 24.0 ≤ BMI < 28.0; normal weight: 18.5 ≤ BMI < 24.0; and underweight: BMI < 18.5.

Smoking status was classified as current smokers (participants were smokers at the time of the survey), ex-smokers (participants were former smokers but had quit at the time of the survey), and never smokers (participants had never smoked). Physical activity status was defined according to the WHO Guidelines on Physical Activity and Sedentary Behaviour for adults aged 18 years and older [13]. Insufficient physical activity was defined as less than 150 min of moderate-intensity activity, less than 75 min of vigorous-intensity activity, or less than 600 MET minutes of the equivalent of moderate-intensity and vigorous-intensity activity in a typical week, and adequate physical activity if any of these requirements was met.

In this study, dietary intakes were specifically assessed, including the consumption of staple foods (such as rice and wheat-based products), red meat, fruits, and vegetables. Staple foods included rice and wheat flour and their products. Red meat referred to pork, beef, and lamb. Fresh fruit excluded fruit juice, dried fruit, preserved fruit, and canned fruit. Vegetables referred to fresh vegetables, excluding dried, pickled vegetables and tubers such as potatoes and cassava. According to the recommendations of the Dietary Guidelines for Chinese Residents (2022) [10] for different daily food intake (200–300 g of cereals, 300–500 g of vegetables, 200–300 g of fruits, and 120–200 g of animal-based food) and the actual intake of different food of the respondents, the average daily staple food intake of rice and flour was divided into four groups: <150 g, 150–249 g, 250–400 g, and >400 g; the average daily intake of red meats was divided into four groups: none, 0–39 g, 40–75 g, and >75 g; the daily intake of fresh vegetables was divided into five groups: <120 g, 120–239 g, 240–299 g, 300–500 g, and >500 g; and the daily intake of fresh fruits was divided into five groups: <25 g, 25–99 g, 100–199 g, 200–350 g, and >350 g.

### 2.6. Statistical Analysis

All data were processed and analyzed using SAS 9.4 software (SAS Institute Inc., Cary, NC, USA). The distribution of drinking status was described as a constituent ratio. Since the four blood lipid levels were not normally distributed, they were expressed as medians (M) and percentiles (P25 and P75). Comparisons of the blood lipid levels of people with different genders and drinking behaviors were compared using the Kruskal–Wallis test, with the pairwise comparison between subgroups conducted using the SCF method.

In view of the large difference in drinking behaviors between men and women, the relationship between different drinking behaviors and dyslipidemia was studied separately by gender. A univariate logistic regression analysis was used to initially examine the association between drinking behaviors and dyslipidemia, followed by multivariate logistic regression. After adjusting for age, region, education level, smoking status, physical activity status, body mass index (BMI), rice and wheat flour intake, red meat intake, fresh vegetable intake, and fresh fruit intake, the effects of different drinking behaviors on dyslipidemia were further analyzed. The results are presented as the odds ratio (OR), 95% confidence interval (CI), along with χ^2^ value and *p* values. Two-sided *p* values of less than 0.05 were considered to indicate statistical significance.

## 3. Results

### 3.1. Characteristics of Research Subjects

This study ultimately included 52,471 research subjects, including 24,028 males (45.8%) and 28,443 females (54.2%); 20,998 in urban areas (40.0%) and 31,473 in rural areas (60.0%) (Table 1). Among the research subjects, the highest proportion of males had a junior high school education level (33.7%), while the highest proportion of females had not received formal education (40.9%). Currently, 52.2% of males currently smoked, compared with only 2.9% of females. The rate of insufficient physical activity was 20.1% for males and 18.0% for females. The rates of overweight and obesity were 35.9% and 13.4% for males, respectively, and 34.7% and 15.2% for females, respectively. The group with the highest proportion of males consuming staple foods (rice/wheat) was the 250–400 g/day category (33.2%), while among females, the 150–249 g/day group was most common (31.3%). Red meat intake was predominantly concentrated in the 0–39 g/day group, accounting for 41.7% of males and 52.2% of females. In contrast, the >75 g/day group comprised 33.5% of males and 22.6% of females. For vegetable intake, the 300–500 g/day category had the highest proportion, with 35.4% of males and 33.9% of females. Fresh fruit consumption was relatively low, with the majority falling into the <25 g/day group (36.4% of males and 31.1% of females).

The abnormal rate of blood lipids in the subjects under investigation was 33.5%, with a higher prevalence in males (37.3%) than in females (30.2%). Among the four types of abnormal blood lipids, the prevalence of low HDL-C was the highest in males (10.%), and the prevalence of high LDL-C was the lowest (4.2%), while the prevalence of hypercholesterolemia was the highest in females (9.2%), and the prevalence of high LDL-C was the lowest (5.9%).

### 3.2. Drinking Status of Chinese Residents

The survey indicates that the prevalence of alcohol consumption among males was 57.5%, much higher than that among women (18.5%) (Table 2). In terms of daily alcohol intake, according to the recommendations of the “Dietary Guidelines for Chinese Residents (2022) [10],” among males, 30.4% were moderate drinkers and 27.1% were excessive drinkers, while among females, moderate drinkers and heavy drinkers account for 16.7% and 1.8%, respectively. Following the guidelines published annually by the WHO in the “Global Status Report on Alcohol and Health [11],” among males, 42.0% were low-risk drinkers, 5.6% were medium-risk drinkers, and 9.9% were high-risk drinkers, while among females, low-risk, medium-risk, and high-risk drinkers account for 17.1%, 0.8%, and 0.6%, respectively.

Regarding the frequency of alcohol consumption, among male drinkers, 30.8% had 1 to 3 drinking episodes per week, 6.7% consume alcohol 4 to 6 times per week, and 1.3% drink daily, while among females, 12.7% drink less than once a week and only 5.8% drink more than once a week.

### 3.3. Blood Lipid Levels of Adult Residents with Different Drinking Behaviors

According to the “Chinese Dietary Guidelines for Residents (2022)” [10], in terms of daily alcohol consumption, among male residents, those who drink excessively have the highest levels of TC (4.84 mmol/L) and HDL-C (1.33 mmol/L) (*p* < 0.0001) (Table 3). Among female residents, those who drink moderately have the lowest levels of TC (4.72 mmol/L) and TG (1.12 mmol/L), (*p* < 0.0001) while those who drink excessively have the highest levels of TC (5.07 mmol/L) and HDL-C (1.45 mmol/L) (*p* < 0.0001).

Based on the WHO’s 2002 International Guide for Monitoring Alcohol Consumption and Related Harm [11], the levels of TC and HDL-C gradually increased among male residents who never drank alcohol, those who were low-risk, those who were moderate-risk, and those who drank alcohol at high-risk (*p* < 0.0001), while female residents who drank alcohol at low risk had the lowest levels of TC, TG, and LDL-C (*p* < 0.0001).

Regarding drinking frequency, among male drinkers, the levels of TC, TG, and HDL-C showed a gradually increasing trend with the increase in drinking frequency (*p* < 0.0001), while in females, those who drank less than once a week had the lowest levels of TC, TG, and LDL-C (*p* < 0.0001). As the frequency of alcohol consumption increases in women, HDL-C levels gradually increase (*p* < 0.0001).

### 3.4. The Relationship Between Male Drinking Behavior and Different Types of Dyslipidemia

The univariate logistic regression analysis showed that compared with non-drinkers, the risk of hypercholesterolemia was significantly higher among male excessive drinkers (OR = 1.50, 95%CI = 1.34–1.68), medium-risk drinkers (OR = 1.33, 95%CI = 1.08–1.63), and high-risk drinkers (OR = 1.85, 95%CI = 1.59–2.14) (Table 4). Similarly, men who consumed alcohol 1–3 times per week (OR = 1.34, 95%CI = 1.20–1.51), 4–6 times/week (OR = 1.44, 95%CI = 1.19–1.73), and daily (OR = 1.76, 95%CI = 1.22–2.53) also had an increased risk of hypercholesterolemia. After full adjustment for confounding factors, the results of the multivariate logistic regression analysis still showed that compared with never drinkers, excessive drinkers (aOR = 1.39, 95%CI = 1.24–1.57), medium -risk drinkers (aOR = 1.24, 95%CI = 1.01–1.53), high-risk drinkers (aOR = 1.67, 95%CI = 1.43–1.95), drinking frequency 1–3 times/week (aOR = 1.27, 95%CI = 1.27; 95%CI = 1.13–1.43), drinking 4 to 6 times per week (aOR = 1.33, 95%CI = 1.10–1.61), and drinking every day (aOR = 1.65, 95%CI = 1.14–2.38) were risk factors for hypercholesterolemia.

A similar pattern was also observed for hypertriglyceridemia. The univariate logistic regression analysis showed that compared with non-drinkers, the risk of hypertriglyceridemia increased with the increase in drinking volume or drinking frequency. This trend persisted after fully adjusting for confounding factors.

Regarding low HDL-C, the univariate logistic regression analysis showed that compared with never drinkers, male moderate drinkers (OR = 0.93, 95%CI = 0.86–0.99) and excessive drinkers (OR = 0.51, 95%CI = 0.47–0.55) had a gradually reduced risk of low HDL-C; low-risk drinkers (OR = 0.84, 95%CI = 0.79–0.90), medium-risk drinkers (OR = 0.45, 95%CI = 0.38–0.52), and high-risk drinkers (OR = 0.38, 95%CI = 0.34–0.44) had a gradually reduced risk of low HDL-C. In terms of frequency of drinking, compared with never drinkers, men who drank alcohol 1–3 times/week (OR = 0.58, 95%CI = 0.54–0.63), 4–6 times/week (OR = 0.62, 95%CI = 0.54–0.71), and every day (OR = 0.48, 95%CI = 0.35–0.66) had a gradually reduced risk of low HDL-C. This pattern was more pronounced in the fully adjusted model, with the results of the multivariate logistic regression analysis showing a significant association. Compared with never drinkers, the aORs for moderate drinking and excessive drinking were 0.82 (95% CI: 0.77–0.89) and 0.48 (95% CI: 0.44–0.52), respectively; the aORs for low-risk drinking, medium-risk drinking, and high-risk drinking were 0.76 (95% CI: 0.71–0.81), 042 (95% CI: 0.36–0.50), and 0.38 (95% CI: 0.33–0.43), respectively; the aORs for drinking less than once a week, 1 to 3 times a week, 4–6 times a week, and daily were 0.90 (95% CI: 0.83–0.97), 0.55 (95% CI: 0.51–0.59), 0.56 (95% CI: 049–0.64), and 0.42 (95% CI: 0.31–0.58).

Regarding the logistic regression analysis, it showed no significant difference in the risk of high LDL-C between any drinking behavior and never drinkers (*p* > 0.05). However, in the fully adjusted model, the results of the multivariate logistic regression analysis showed that compared with never drinkers, the risk of high LDL-C was lower in excessive drinkers (aOR = 0.86, 95% CI: 0.77–0.97), medium-risk drinkers (aOR = 0.80, 95% CI: 0.65–0.99), high-risk drinkers (aOR = 0.83, 95% CI: 0.70–0.97), and drinkers who consumed alcohol 1 to 3 times week (aOR = 0.89, 95% CI: 0.79–0.99).

### 3.5. The Relationship Between Female Drinking Behavior and Different Types of Dyslipidemia

The univariate logistic regression analysis showed that among women, compared with never drinkers, moderate drinkers (OR = 0.82, 95%CI = 0.73–0.92), low-risk drinkers (OR = 0.83, 95%CI = 0.74–0.92), and those drinking less than once a week (OR = 0.78, 95%CI = 0.68–0.89) had a lower risk of hypercholesterolemia (Table 5). However, after full adjustment for confounding factors, the multivariate logistic regression analysis showed no significant difference in the risk of hypercholesterolemia between alcohol drinkers and non-drinkers (*p* > 0.05).

Regarding the risk of hypertriglyceridemia, the univariate logistic regression analysis showed that compared with never drinkers, female moderate drinkers (OR = 0.79, 95%CI = 0.72–0.87), low-risk drinkers (OR = 0.80, 95%CI = 0.72–0.88), drinking frequency < 1 time/week (OR = 0.82, 95%CI = 0.74–0.92), and ≥1 time/week (OR = 0.77, 95%CI = 0.65–0.90) were at a lower risk of hypertriglyceridemia. After fully adjusting for confounding factors, the multivariate logistic regression analysis results still showed that compared with never drinkers, women with moderate alcohol consumption (aOR = 0.85, 95%CI = 0.77–0.94), low-risk alcohol consumption (aOR = 0.86, 95%CI = 0.78–0.94), and consumption ≥ 1 time/week (aOR = 0.74, 95%CI = 0.63–0.87) had a lower risk of hypertriglyceridemia.

For low HDL-C, the univariate logistic regression analysis showed that compared with never drinkers, moderate drinkers (OR = 0.84, 95%CI = 0.77–0.92) and excessive drinkers (OR = 0.47, 95%CI = 0.34–0.65) had a gradually reduced risk of low HDL-C in women. Low-risk drinkers (OR = 0.84, 95%CI = 0.76–0.92), medium-risk drinkers (OR = 0.52, 95%CI = 0.32–0.85), and high-risk drinkers (OR = 0.25, 95%CI = 0.12–0.53) had a gradually reduced risk of low HDL-C. In terms of drinking frequency, the risk of low HDL-C decreased in women who drank to more than 1 time/week (OR = 0.59, 95%CI = 0.50–0.70) compared with never drinkers. This pattern existed after fully adjusting for confounding factors and performing a multivariate logistic regression analysis. Compared with never drinkers, the aORs for moderate drinking and excessive drinking were 0.84 (95%CI = 0.77–0.93) and 0.47 (95%CI = 0.34–0.66), respectively; the aORs for low-risk drinking, medium-risk drinking, and high-risk drinking were 0.84 (95% = 0.76–0.92), 0.52 (95%CI = 0.32–0.84), and 0.26 (95%CI = 0.12–0.55), respectively; the aOR for drinking ≥ 1 times/week was 0.59 (95%CI = 0.50–0.70).

The univariate logistic regression analysis showed that compared with never drinkers, moderate drinkers (OR = 0.80, 95%CI = 0.72–0.89), low-risk drinkers (OR = 0.82, 95%CI = 0.74–0.91), and those who drank less than once a week (OR = 0.77, 95%CI = 0.68–0.87) had a lower risk of high LDL-C. However, after fully adjusting for confounding factors, the multivariate logistic regression analysis showed that there was no significant difference in the risk of high LDL-C between alcohol drinkers and non-drinkers (*p* > 0.05).

## 4. Discussion

The 2019 ESC/EAS Guidelines for the Management of Dyslipidaemias emphasize that LDL-C levels exhibit a linear relationship with cardiovascular risk, and reducing LDL-C is a key strategy for preventing cardiovascular disease [14]. Dyslipidemia is not only a critical risk factor for cardiovascular diseases but also a major modifiable and reversible one. Research has demonstrated that alcohol consumption influences plasma lipoprotein metabolism [15], and some studies have shown that alcohol consumption is an independent risk factor for dyslipidemia [16].

We observed inconsistent impacts of alcohol intake across different lipid markers. This study found that male excessive drinkers and high-risk drinkers exhibited the highest levels of total TC and TG among all participants. The multivariate logistic regression analysis, after adjusting for potential confounders including region, education level, smoking status, physical activity, body mass index (BMI), consumption of rice and wheat flour, red meat intake, and fresh vegetable and fruit consumption, revealed that male alcohol consumers had significantly higher risks of developing hypercholesterolemia (aOR range: 1.24–1.67) and hypertriglyceridemia (aOR range: 1.10–1.52) compared with non-drinkers. The risk increased with higher alcohol consumption and frequency. These findings are consistent with other relevant studies. A study conducted by the chronic disease and risk factor surveillance system across 31 provinces (including autonomous regions and municipalities) in China revealed that as alcohol consumption increases and the frequency of alcohol consumption rises, the risk of hypercholesterolemia (aOR range: 1.16–1.36) and hypertriglyceridemia (aOR range: 1.14–1.64) among male drinkers gradually increases [17]. A cohort study based on a Korean community revealed that alcohol consumption is a risk factor for hypertriglyceridemia in men, with current drinkers showing elevated triglyceride levels compared with those who never drank [18]. Data from the Korean National Health and Nutrition Examination Survey (KNHANES) indicated that the frequency of alcohol consumption was associated with elevated triglyceride levels in Korean men aged 20 to 79 years [19]. A prospective cohort study in the Mediterranean region showed that the risk of hypertriglyceridemia in drinkers who consumed seven glasses of alcohol per week was 2.07 times that of non-drinkers [20]. The Paris-Ile-derance City Cohort Study showed that total cholesterol levels were positively correlated with alcohol consumption [21]. The total cholesterol levels were the lowest in those who never consumed alcohol and the highest in those who abused alcohol. The hypertriglyceridemia caused by alcohol consumption may be related to the increase in very low-density lipoprotein caused by alcohol, the impairment of lipolysis, and the increase in free fatty acids from adipose tissue to the liver [22,23,24]. Those who consume alcohol are more likely to adopt unhealthy diets, such as those high in salt and fat, and a significant proportion of drinkers engage in unhealthy lifestyles, including smoking [25]. Dietary saturated fatty acids can promote the synthesis of TC, leading to increased TC levels [26]. Studies have shown that individuals who both smoke and drink concurrently have a higher risk of dyslipidemia compared with those who only smoke [27].

However, there are also inconsistent findings across studies. A cross-sectional study of Poland–Norway reported no relationship between alcohol consumption and triglyceride levels [28]. Additionally, Asians have been shown to exhibit significantly higher plasma triglyceride concentrations compared with other ethnic groups, which may be attributable to genetic factors [29].

This study revealed an intriguing gender-specific pattern: female moderate drinkers and low-risk drinkers exhibited the lowest plasma TG levels among all groups (all *p* < 0.05). The adjusted multivariate analysis further demonstrated that, compared with abstainers, women with moderate alcohol consumption, low-risk drinking patterns, or weekly alcohol intake (≥1 drink/week) had significantly lower risks of developing hypertriglyceridemia (aOR range: 0.63–0.94). Notably, this protective association was not observed in other drinking patterns. The protective effect of moderate alcohol consumption on TG levels in women may be attributed to estrogen–alcohol synergism and sex-specific alcohol metabolism. Estrogen upregulates lipoprotein lipase (LPL) activity, enhancing fatty acid clearance and reducing plasma TG levels [30]. Red wine consumption is more prevalent among Chinese women than men [31]. Red wine polyphenols may activate AMPK signaling, suppressing hepatic de novo lipogenesis and VLDL assembly, thereby decreasing TG secretion [32]. Moderate alcohol potentiates the estrogen-mediated regulation of TG metabolism in adipocytes [33,34]. Higher gastric alcohol dehydrogenase (ADH) activity in males accelerates the first-pass metabolism of low-dose alcohol, preventing its accumulation to biologically effective concentrations. In contrast, women’s lower ADH activity permits sustained exposure at equivalent doses, potentially amplifying metabolic effects [35,36,37].

This study identified a consistent alcohol–HDL-C association across genders: both males and females demonstrated progressively elevated HDL-C levels with increasing alcohol consumption. Logistic regression analysis confirmed this dose–response relationship, revealing that compared with abstainers, all drinking groups showed significantly reduced risks of low HDL-C levels (*p* < 0.0001), with risk reduction magnitudes correlating positively with both alcohol quantity and frequency. These associations remained statistically significant after full adjustment for potential confounders, including demographic, lifestyle, and dietary factors.

In both men and women, the risk of low HDL-C levels decreased with increasing alcohol consumption volume or frequency compared with non-drinkers. This reduction in risk remained significant after adjusting for potential confounders. Similar findings have been reported in other studies [38]. A cross-sectional study in Poland demonstrated that current alcohol consumption was associated with higher HDL-C in both sexes [28]. The Paris-Ile-de-France Urban cohort study also revealed a robust association between alcohol intake and plasma HDL-C levels in both genders [21]. Korean men who consume alcohol 2–3 times per week or more, and women who drink 2–4 times per month or more, exhibit a lower risk of low HDL-C compared with those who never drink [19]. Individuals who consume alcohol ≥ seven times per week have a 0.63-fold lower risk of low HDL-C compared with non-drinkers [20]. The potential mechanism by which alcohol affects HDL-C may involve reducing the activity of cholesteryl ester transfer protease, leading to increased serum HDL-C levels [39,40], or by enhancing hepatic production and/or the transport rate of HDL apolipoproteins apoA-I and apoA-II. Additionally, alcohol may increase cellular cholesterol efflux and plasma cholesterol esterification [28,41,42,43].

It is crucial to note that these studies primarily focused on changes in HDL-C quantity rather than HDL quality and function between drinkers and non-drinkers. HDL-C possesses potent antioxidant and anti-inflammatory activities and plays a critical role in reverse cholesterol transport, which can inhibit the initiation of atherosclerosis. Maintaining optimal HDL-C quality is essential for its functionality [44,45]. A study of Korean women found that even small amounts of alcohol can lead to reduced HDL-C particle size and increased aggregation, impairing HDL-C quality and function, particularly in women with chronic alcohol consumption [45]. Therefore, although many studies have shown that alcohol consumption can increase the levels of HDL-C, alcohol may also impair the function of HDL-C [45,46]. Thus, the effect of alcohol on HDL-C levels should not be interpreted simply as a protective effect.

In men, after adjusting for potential confounders, excessive drinking, medium-risk drinking, and high-risk drinking were associated with a greater than 10% reduction in the risk of elevated LDL-C levels. This association was not observed in women. The Paris-Ile-de-France Urban cohort study demonstrated that women with moderate alcohol consumption exhibited lower fasting triglyceride and LDL-C levels [21]. A prospective randomized trial indicated a 16% reduction in LDL-C in the wine group compared with other groups after three months of intervention [47]. The reduction in LDL-C may be attributed to the type of wine consumed, particularly the polyphenols in red wine which can reduce lipid oxidation [48]. However, further research is required to substantiate this claim.

This study utilized a large, nationally representative sample and adjusted for all possible confounding factors, thereby providing a robust reflection of the relationship between drinking behavior and dyslipidemia. These findings carry important public health implications, as they may guide people toward rational drinking and reduce future alcohol-related health risks. However, this study has limitations. Firstly, alcohol consumption and dietary data were collected via self-reported recall over the previous year, which may introduce recall bias. Secondly, as a cross-sectional study, it cannot establish causality between alcohol use and dyslipidemia. Future studies should use prospective cohorts to explore the relationship between alcohol consumption and dyslipidemia causality.

In China, alcohol consumption is deeply embedded in social customs, particularly during family and friends’ gatherings, where inebriation is often humorously encouraged. However, this culturally normalized practice carries substantial health hazards. While our nationwide cross-sectional study and existing evidence indicate that alcohol use may correlate with certain favorable lipid alterations including elevated HDL-C levels in both genders and reduced TG levels among moderate female drinkers, the current elevation of HDL-C level by alcohol consumption cannot be definitely interpreted as a protective effect. More importantly, alcohol intake demonstrates a pronounced adverse effect on male lipid profiles, significantly elevating both TG and TC levels. In light of these findings and the equivocal nature of potential benefits, we strongly advise against endorsing alcohol consumption as a health-promoting behavior in Chinese adults.

## 5. Conclusions

The effects of different drinking behaviors on different lipid levels are inconsistent, and there are significant gender differences. Our findings indicate the following: Men who were excessive drinkers, medium-risk drinkers, high-risk drinkers, and those who drank more than once a week had a higher risk of hypercholesterolemia and hypertriglyceridemia, but a lower risk of low HDL-C and high LDL-C. Women who drank moderately had a lower risk of hypertriglyceridemia. Any level of alcohol consumption and a drinking frequency of more than once per week were associated with a lower risk of low HDL-C in women, while any drinking behavior in women was not associated with the risk of hypercholesterolemia and high LDL-C.

## Figures and Tables

**Table 1:** Sample distribution of adult residents with different characteristics in China (*n*, %).

Characteristics	Male		Female		Total	
	*n*	Percentage (%)	*n*	Percentage (%)	*n*	Percentage (%)
Age group (Years)						
18–44	5735	23.9	7487	26.3	13,222	25.2
45–59	8666	36.1	10,944	38.5	19,610	37.4
60–69	6262	26.1	6859	24.1	13,121	25.0
70–79	2737	11.4	2573	9.1	5310	10.1
≥80	628	2.6	580	2.0	1208	2.3
Residential area						
Urban	9460	39.4	11,538	40.6	20,998	40.0
Rural	14,568	60.6	16,905	59.4	31,473	60.0
Educational level *						
NS	5216	21.7	11,625	40.9	16,841	32.1
PS	5532	23.0	5571	19.6	11,103	21.2
JS	8092	33.7	6923	24.3	15,015	28.6
HS	3500	14.6	2655	9.3	6155	11.7
TS	1004	4.2	1051	3.7	2055	3.9
C or above	684	2.9	618	2.2	1302	2.5
Smoking status						
Current	12,538	52.2	829	2.9	13,367	25.5
Former	3555	14.8	223	0.8	3778	7.2
Never	7935	33.0	27,391	96.3	35,326	67.3
Physical activity status						
Insufficient	4838	20.1	4616	16.2	9454	18.0
Sufficient	19,190	79.9	23,827	83.8	43,017	82.0
Weight status						
Obesity	3226	13.4	4326	15.2	7552	14.4
Overweight	8624	35.9	9865	34.7	18,489	35.2
Normal	11,316	47.1	13,099	46.1	24,415	46.5
Underweight	862	3.6	1153	4.1	2015	3.8
Daily average intake of rice and wheat flour staple food (g)						
<150	3135	13.1	6410	22.5	9545	18.2
150–249	5933	24.7	8895	31.3	14,828	28.3
250–400	7975	33.2	8484	29.8	16,459	31.4
>400	6985	29.1	4654	16.4	11,639	22.2
Daily average intake of red meat (g)						
0	513	2.1	1171	4.1	1684	3.2
0–39	10,022	41.7	14,832	52.2	24,854	47.4
40–75	5443	22.7	6025	21.2	11,468	21.9
>75	8050	33.5	6415	22.6	14,465	27.6
Daily fresh vegetable intake (g)						
<120	5421	22.6	6933	24.4	12,354	23.5
120–239	5904	24.6	7408	26.1	13,312	25.4
240–299	1047	4.4	1247	4.4	2294	4.4
300–500	8509	35.4	9639	33.9	18,148	34.6
>500	3147	13.1	3216	11.3	6363	12.1
Daily fresh fruit intake (g)						
<25	8742	36.4	8843	31.1	17,585	33.5
25–99	7465	31.1	8391	29.5	15,856	30.2
100–199	4268	17.8	5982	21.0	10,250	19.5
200–350	2836	11.8	4038	14.2	6874	13.1
>350	717	3.0	1189	4.2	1906	3.6
Prevalence of dyslipidemia						
Hyperlipidemia	8969	37.3	8595	30.2	17,564	33.5
Hypercholesterolemia	1770	7.4	26.6	9.2	4376	8.3
Hypertriglyceridemia	4142	7.9	3833	7.3	7975	15.2
Low HDL-C	5578	10.6	4051	7.7	9629	18.4
High LDL-C	2186	4.2	3072	5.9	5253	10.0
Total	24,028	46	28,443	54	52,471	100

* Educational level: NS (not receiving formal education from school), PS (primary school), JS (junior high school), HS (high school), TS (technical school), C (college).

**Table 2:** Alcohol consumption of adult residents in China (*n*, %).

Characteristics	Male		Female	
	*n*	Percentage (%)	*n*	Percentage (%)
Alcohol Consumption (China Classification)				
Never	10,205	42.5	23,184	81.5
Moderate	7309	30.4	4751	16.7
Excessive	6514	27.1	508	1.8
Alcohol Consumption (WHO Classification)				
Never	10,205	42.5	23,184	81.5
Low risk	10,101	42.0	4872	17.1
Medium risk	1351	5.6	218	0.8
High risk	2371	9.9	169	0.6
Alcohol Consumption Frequency (Times/Week)				
Never	10,205	42.5	23,184	81.5
<1	4506	18.8	3614	12.7
1–3	7402	30.8	1447	5.1
4–6	1605	6.7	175	0.6
≥7	310	1.3	23	0.1
Total	24,028	45.8	28,443	54.2

**Table 3:** Blood lipid levels of adult residents aged 18 and above in China with different drinking behaviors (mmol/L).

Characteristics		TC M (P25, P75)	TG M (P25, P75)	HDL-C M (P25, P75)	LDL-C M (P25, P75)
Male	Alcohol Consumption (China Classification)				
	Never	4.61 (4.02, 5.26) ^a^	1.18 (0.83, 1.77) ^a^	1.17 (0.98, 1.39) ^a^	2.89 (2.35, 3.46)
	Moderate	4.67 (4.09, 5.29) ^b^	1.26 (0.87, 1.92) ^b^	1.17 (0.99, 1.40) ^a^	2.92 (2.40, 3.45)
	Excessive	4.84 (4.27, 5.54) ^c^	1.25 (0.83, 2.01) ^b^	1.33 (1.09, 1.61) ^b^	2.90 (2.37, 3.44)
	χ^2^	242.324	55.213	901.271	5.896
	*p*	<0.0001	<0.0001	<0.0001	0.05
	Alcohol Consumption (WHO Classification)				
	Never	4.61 (4.02, 5.26) ^a^	1.18 (0.83, 1.77) ^a^	1.17 (0.98, 1.39) ^a^	2.89 (2.35, 3.46)
	Low risk	4.69 (4.13, 5.31) ^b^	1.26 (0.86, 1.93) ^b^	1.20 (1.01, 1.43) ^b^	2.92 (2.40, 3.45) ^a^
	Medium risk	4.83 (4.23, 5.42) ^c^	1.23 (0.81, 1.97)	1.32 (1.10, 1.58) ^c^	2.88 (2.37, 3.43)
	High risk	4.95 (4.35, 5.56) ^d^	1.26 (0.82, 2.08) ^b^	1.41 (1.15, 1.71) ^d^	2.88 (2.33, 3.44) ^b^
	χ^2^	252.428	56.575	929.569	12.916
	*p*	<0.0001	<0.0001	<0.0001	0.0048
	Alcohol Consumption Frequency (Times/Week)				
	Never	4.61 (4.02, 5.26) ^a^	1.18 (0.83, 1.77) ^a^	1.17 (0.98, 1.39) ^a^	2.89 (2.35, 3.46)
	<1	4.64 (4.07, 5.26) ^a^	1.28 (0.88, 1.94) ^b^	1.15 (0.98, 1.37) ^a^	2.91 (2.42, 3.45)
	1–3	4.80 (4.23, 5.41) ^b^	1.22 (0.82, 1.93) ^c^	1.29 (1.07, 1.56) ^b^	2.91 (2.37, 3.45)
	4–6	4.81 (4.18, 5.41) ^b^	1.29 (0.85, 2.08) ^b^	1.28 (1.06, 1.55) ^b^	2.89 (2.35, 3.45)
	≥7	4.90 (4.31, 5.42) ^b^	1.36 (0.90, 2.16) ^b^	1.31 (1.09, 1.51) ^b^	2.98 (2.47, 3.45)
	χ^2^	210.560	72.881	792.797	5.032
	*p*	<0.0001	<0.0001	<0.0001	0.28
Female	Alcohol Consumption (China Classification)				
	Never	4.80 (4.18, 5.48) ^a^	1.21 (0.83, 1.78) ^a^	1.29 (1.09, 1.51) ^a^	2.96 (2.42, 3.55) ^a^
	Moderate	4.72 (4.11, 5.40) ^b^	1.12 (0.79, 1.63) ^b^	1.31 (1.12, 1.52) ^b^	2.89 (2.35, 3.49) ^b^
	Excessive	5.07 (4.47, 5.66) ^c^	1.23 (0.84, 1.72) ^a^	1.45 (1.21, 1.68) ^c^	3.05 (2.48, 3.58) ^a^
	χ^2^	56.591	59.999	113.545	30.132
	*p*	<0.0001	<0.0001	<0.0001	<0.0001
	Alcohol Consumption (WHO Classification)				
	Never	4.80 (4.18, 5.48) ^a^	1.21 (0.83, 1.78) ^a^	1.29 (1.09, 1.51) ^a^	2.96 (2.42, 3.55) ^a^
	Low risk	4.72 (4.12, 5.41) ^b^	1.12 (0.79, 1.64) ^b^	1.31 (1.12, 1.53) ^b^	2.89 (2.36, 3.49) ^b^
	Medium risk	5.13 (4.51, 5.63) ^c^	1.19 (0.83, 1.65)	1.46 (1.23, 1.66) ^c^	3.06 (2.43, 3.61)
	High risk	5.08 (4.48, 5.65) ^c^	1.17 (0.76, 1.85)	1.51 (1.26, 1.75) ^c^	2.94 (2.43, 3.46)
	χ^2^	47.366	55.481	127.764	24.165
	*p*	<0.0001	<0.0001	<0.0001	<0.0001
	Alcohol Consumption Frequency (Times/Week)				
	Never	4.80 (4.18, 5.48) ^a^	1.21 (0.83, 1.78) ^a^	1.29 (1.09, 1.51) ^a^	2.96 (2.42, 3.55) ^a^
	<1	4.69 (4.08, 5.37) ^b^	1.11 (0.79, 1.63) ^b^	1.30 (1.11, 1.51) ^a^	2.87 (2.33, 3.47) ^b^
	≥1	4.90 (4.26, 5.53) ^c^	1.16 (0.80, 1.66) ^b^	1.38 (1.17, 1.61) ^b^	2.98 (2.43, 3.54) ^a^
	χ^2^	54.084	56.060	117.476	32.914
	*p*	<0.0001	<0.0001	<0.0001	<0.0001

M (P25, P75): median (percentile 25, percentile 75). Different letters indicate a statistically significant difference between the two groups (*p* < 0.05); the same letters indicate no statistically significant difference between two groups (*p* > 0.05).

**Table 4:** The relationship between different drinking behaviors and dyslipidemia among adult male residents aged 18 and above in China.

Characteristics	High TC		High TG		Low HDL-C		High LDL-C	
	OR (95%CI)	aOR (95%CI)	OR (95%CI)	aOR (95%CI)	OR (95%CI)	aOR (95%CI)	OR (95%CI)	aOR (95%CI)
Alcohol Consumption (China Classification)								
Never	1.00	1.00	1.00	1.00	1.00	1.00	1.00	1.00
Moderate	1.00 (0.89–1.13)	1.00 (0.88–1.13)	1.22 (1.12–1.32) *	1.05 (0.97–1.15)	0.93 (0.86–0.99) *	0.82 (0.77–0.89) *	0.94 (0.85–1.05)	0.94 (0.84–1.04)
Excessive	1.50 (1.34–1.68) *	1.39 (1.24–1.57) *	1.49 (1.38–1.62) *	1.35 (1.24–1.47) *	0.51 (0.47–0.55) *	0.48 (0.44–0.52) *	0.91 (0.82–1.02)	0.86 (0.77–0.97) *
χ^2^	59.966	38.121	94.423	52.729	294.159	301.349	2.943	6.4
*p*	<0.0001	<0.0001	<0.0001	<0.0001	<0.0001	<0.0001	0.23	0.0408
Alcohol Consumption (WHO Classification)								
Never	1.00	1.00	1.00	1.00	1.00	1.00	1.00	1.00
Low risk	1.08 (0.97–1.21)	1.06 (0.95–1.19)	1.27 (1.18–1.38) *	1.10 (1.02–1.19) *	0.84 (0.79–0.90) *	0.76 (0.71–0.81) *	0.95 (0.86–1.04)	0.93 (0.85–1.03)
Medium risk	1.33 (1.08–1.63) *	1.24 (1.01–1.53) *	1.41 (1.22–1.63) *	1.29 (1.11–1.50) *	0.45 (0.38–0.52) *	0.42 (0.36–0.50) *	0.84 (0.69–1.04)	0.80 (0.65–0.99) *
High risk	1.85 (1.59–2.14) *	1.67 (1.43–1.95) *	1.64 (1.47–1.84) *	1.52 (1.35–1.71) *	0.38 (0.34–0.44) *	0.38 (0.33–0.43) *	0.9 (0.76–1.05)	0.83 (0.70–0.97) *
χ^2^	70.722	46.091	93.641	53.746	281.063	288.675	4.161	8.257
*p*	<0.0001	<0.0001	<0.0001	<0.0001	<0.0001	<0.0001	0.2446	0.041
Alcohol Consumption Frequency (Times/Week)								
Never	1.00	1.00	1.00	1.00	1.00	1.00	1.00	1.00
<1	0.95 (0.82–1.10)	0.95 (0.82–1.10)	1.24 (1.13–1.36) *	1.06 (0.96–1.17)	1.02 (0.94–1.10)	0.90 (0.83–0.97) *	0.93 (0.82–1.05)	0.93 (0.82–1.05)
1–3	1.34 (1.20–1.51) *	1.27 (1.13–1.43) *	1.34 (1.24–1.45) *	1.22 (1.12–1.33) *	0.58 (0.54–0.63) *	0.55 (0.51–0.59) *	0.92 (0.83–1.02)	0.89 (0.79–0.99) *
4–6	1.44 (1.19–1.73) *	1.33 (1.10–1.61) *	1.62 (1.42–1.84) *	1.36 (1.19–1.55) *	0.62 (0.54–0.71) *	0.56 (0.49–0.64) *	0.92 (0.76–1.11)	0.87 (0.72–1.05)
≥7	1.76 (1.22–2.53) *	1.65 (1.14–2.38) *	1.64 (1.25–2.16) *	1.38 (1.05–1.83) *	0.48 (0.35–0.66) *	0.42 (0.31–0.58) *	1.22 (0.86–1.75)	1.16 (0.81–1.66)
χ^2^	47.971	31.406	87.338	35.667	270.478	281.48	5.012	7.143
*p*	<0.0001	<0.0001	<0.0001	<0.0001	<0.0001	<0.0001	0.2861	0.1285

aOR: OR values after controlling for age, region, education level, smoking status, physical activity status, body mass index (BMI), rice and wheat flour intake, red meat intake, fresh vegetable intake, and fresh fruit intake. * The 95% confidence interval does not include 1, and the difference between the two groups is significant (*p* < 0.05).

**Table 5:** The relationship between different drinking behaviors and dyslipidemia among adult female residents aged 18 and above in China.

Characteristics	High TC		High TG		Low HDL-C		High LDL-C	
	OR (95%CI)	aOR (95%CI)	OR (95%CI)	aOR (95%CI)	OR (95%CI)	aOR (95%CI)	OR (95%CI)	aOR (95%CI)
Alcohol Consumption (China Classification)								
Never	1.00	1.00	1.00	1.00	1.00	1.00	1.00	1.00
Moderate	0.82 (0.73–0.92) *	0.94 (0.83–1.05)	0.79 (0.72–0.87) *	0.85 (0.77–0.94) *	0.84 (0.77–0.92) *	0.84 (0.77–0.93) *	0.80 (0.72–0.89) *	0.90 (0.81–1.01)
Excessive	1.20 (0.90–1.58)	1.05 (0.79–1.39)	0.94 (0.73–1.22)	0.89 (0.68–1.15)	0.47 (0.34–0.65) *	0.47 (0.34–0.66) *	1.13 (0.87–1.48)	1.00 (0.76–1.32)
χ^2^	13.6989	1.3629	22.3474	10.4013	32.2812	30.8687	17.6285	3.3331
*p*	0.0011	0.5059	<0.0001	0.0055	<0.0001	<0.0001	0.0001	0.1889
Alcohol Consumption (WHO Classification)								
Never	1.00	1.00	1.00	1.00	1.00	1.00	1.00	1.00
Low risk	0.83 (0.74–0.92) *	0.94 (0.83–1.05)	0.80 (0.72–0.88) *	0.86 (0.78–0.94) *	0.84 (0.76–0.92) *	0.84 (0.76–0.92) *	0.82 (0.74–0.91) *	0.92 (0.82–1.02)
Medium risk	1.19 (0.78–1.83)	1.09 (0.70–1.68)	0.73 (0.47–1.13)	0.7 (0.45–1.09)	0.52 (0.32–0.85) *	0.52 (0.32–0.84) *	0.99 (0.65–1.52)	0.90 (0.58–1.39)
High risk	1.30 (0.81–2.07)	1.07 (0.67–1.72)	1.18 (0.78–1.78)	1.09 (0.72–1.65)	0.25 (0.12–0.53) *	0.26 (0.12–0.55) *	1.01 (0.63–1.64)	0.85 (0.53–1.39)
χ^2^	13.3958	1.5127	24.1004	12.2052	33.266	31.7914	13.7376	2.937
*p*	0.0039	0.6793	<0.0001	0.0067	<0.0001	<0.0001	0.0033	0.4014
Alcohol Consumption Frequency (Times/Week)								
Never	1.00	1.00	1.00	1.00	1.00	1.00	1.00	1.00
<1	0.78 (0.68–0.89) *	0.94 (0.82–1.07)	0.82 (0.74–0.92) *	0.92 (0.82–1.02)	0.90 (0.82–1.00)	0.91 (0.82–1.01)	0.77 (0.68–0.87) *	0.91 (0.81–1.04)
≥1	1.03 (0.87–1.21)	0.97 (0.82–1.16)	0.77 (0.65–0.90) *	0.74 (0.63–0.87) *	0.59 (0.50–0.70) *	0.59 (0.50–0.70) *	0.97 (0.83–1.14)	0.91(0.78–1.08)
χ^2^	14.2258	0.9825	22.0628	14.6089	38.9696	38.3608	17.2455	2.8535
*p*	0.002	0.6119	<0.0001	0.0007	<0.0001	<0.0001	0.0006	0.2401

aOR: OR values after controlling for age, region, education level, smoking status, physical activity status, body mass index (BMI), rice and wheat flour intake, red meat intake, fresh vegetables intake and fresh fruit intake. * The 95% confidence interval does not include 1, and the difference between the two groups is significant (*p* < 0.05).

## Data Availability

The data used in this study are not publicly available due to privacy.

## References

[1] World Health Organization (2018). Global Status Report on Alcohol and Health 2018.

[2] Ference B.A., Ginsberg H.N., Graham I., Ray K.K., Packard C.J., Bruckert E., Hegele R.A., Krauss R.M., Rfaal F.J., Schunkert H. (2017). Low-density lipoproteins cause atherosclerotic cardiovascular disease. 1. Evidence from genetic, epidemiologic, and clinical studies. A consensus statement from the European Atherosclerosis Society Consensus Panel. Eur. Heart J..

[3] Fulcher J., O’Connell R., Voysey M., Emberson E., Blackwell L., Mihaylova B., Laboratory Medicine Society of Chinese Medical Association, Branch of Laboratory Physicians of Chinese Medical Doctor Association, Lipids and Lipoproteins Committee, Cholesterol Treatment Trialists’ (CTT) Collaboration (2022). Chinese Clinical Lipid Testing Guidelines. Chin. J. Lab. Med..

[4] Gotto A.M., Brinton E.A. (2004). Assessing low levels of high-density lipoprotein cholesterol as a risk factor in coronary heart disease: A working group report and update. J. Am. Coll. Cardiol..

[5] Navarese E.P., Robinson J.G., Kowalewski M., Kołodziejczak M., Andreotti F., Bliden K. (2018). Association Between Baseline LDL-C Level and Total and Cardiovascular Mortality After LDL-C Lowering: A Systematic Review and Meta-analysis. JAMA.

[6] Fulcher J., Oçonnel R., Voysey M., Emberson J., Blackwell L., Mihaylove B., Simes J., Collins R., Kirby A., Cholesterol Treatment Trialists’ (CTT) Collaboration (2015). Efficacy and safety of LDL-lowering therapy among men and women: Meta-analysis of individual data from 174,000 participants in 27 randomised trials. Lancet.

[7] Joint Committee of the Chinese Guidelines for Lipid Management (2023). Chinese guidelines for lipid management (2023). Chin. J. Cardiol..

[8] National Health Commission of China, Department of Chronic Disease and Prevention Bureau (2021). Report on the Nutritional and Chronic Disease Status of Chinese Residents (2020).

[9] Yu D., Zhao L., Zhang J., Yang Z., Yang L., Huang J. (2021). China Nutrition and Health Surveys (1982–2017). China CDC Wkly..

[10] Chinese Nutrition Society (2022). Dietary Guidelines for Chinese Residents (2022).

[11] WHO (2002). International Guide for Monitoring Alcohol Consumption and Related Harm.

[12] (2013). National Health Commission of the People’s Republic of China. Adult Weight Judgment.

[13] Bull F.C., Al-Ansari S.S., Biddle S., Borodulin K., Buman M.P., Cardon G. (2020). World Health Organization 2020 guidelines on physical activity and sedentary behaviour. Br. J. Sports Med..

[14] The Task Force for the management of dyslipidaemias of the European Society of Cardiology (ESC), European Atherosclerosis Society (EAS) (2019). 2019 ESC/EAS guidelines for the management of dyslipidaemias: Lipid modification to reduce cardiovascular risk. Eur. Heart J..

[15] Frohlich J.J. (1996). Effects of alcohol on plasma lipoprotein metabolism. Clin. Chim. Acta.

[16] Chen Y., Du J., Zhou N., Song Y., Wang W., Hong X. (2023). Prevalence, awareness, treatment and control of dyslipidaemia and their determinants: Results from a population-based survey of 60,283 residents in eastern China. BMJ Open.

[17] Hu C.H., Zhang M., Li C., Zhao Z.P., Zhang X., Huang Z.J. (2020). Study on the relationship between drinking behavior and dyslipidemia among Chinese adults. Dis. Surveill..

[18] Kim S.K., Hong S.H., Chung J.H., Cho K.B. (2017). Association Between Alcohol Consumption and Metabolic Syndrome in a Community-Based Cohort of Korean Adults. Med. Sci. Monit. Int. Med. J. Exp. Clin. Res..

[19] Oh S.S., Kim W., Han K.T., Park E.C., Jang S.I. (2018). Alcohol consumption frequency or alcohol intake per drinking session: Which has a larger impact on the metabolic syndrome and its components?. Alcohol.

[20] Barrio-Lopez M.T., Bes-Rastrollo M., Sayon-Orea C., Garcia-Lopez M., Fernandez-Montero A., Gea A., Martinez-Gonzalez M.A. (2013). Different types of alcoholic beverages and incidence of metabolic syndrome and its components in a Mediterranean cohort. Clin. Nutr..

[21] Hansel B., Thomas F., Pannier B., Bean K., Kontush A., Chapman M.J. (2010). Relationship between alcohol intake, health and social status and cardiovascular risk factors in the Urban Paris-Ile-de-France Cohort: Is the cardioprotective action of alcohol a myth?. Eur. J. Clin. Nutr..

[22] Shen Z., Munker S., Wang C., Xu L., Ye H., Chen H., Xu G., Zhang H., Chen L., Yu C. (2014). Association between alcohol intake, overweight, and serum lipid levels and the risk analysis associated with the development of dyslipidemia. J. Clin. Lipidol..

[23] Klop B., do Rego A.T., Cabezas M.C. (2013). Alcohol and plasma triglycerides. Curr. Opin. Lipidol..

[24] Capurso N.A., Petrakis I. (2016). Dyslipidemia associated with heavy alcohol use. Am. J. Addict..

[25] Park H., Kim K. (2012). Alcohol, and alcoholism, Association of alcohol consumption with lipid profile in hypertensive men. Alcohol Alcohol..

[26] Alcohol I.W.J. (2013). Associations between heavy alcohol drinking and lipid-related indices in middle-aged men. Alcohol.

[27] Li X.X., Zhao Y., Huang L.X., Xu H.X., Liu X.Y., Yang J.J. (2018). Effects of smoking and alcohol consumption on lipid profile in male adults in northwest rural China. Public Health.

[28] Suliga E., Kozieł D., Ciesla E., Rebak D., Głuszek-Osuch M., Głuszek S. (2019). Consumption of Alcoholic Beverages and the Prevalence of Metabolic Syndrome and Its Components. Nutrients.

[29] Park S., Kang S. (2020). Nutrition, and Dietetics, Alcohol, Carbohydrate, and Calcium Intakes and Smoking Interactions with APOA5 rs662799 and rs2266788 were Associated with Elevated Plasma Triglyceride Concentrations in a Cross-Sectional Study of Korean Adults. J. Acad. Nutr. Diet..

[30] Luo F., Huang W.-Y., Guo Y., Ruan G.-Y., Peng R., Li X.-P. (2017). 17β-estradiol lowers triglycerides in adipocytes via estrogen receptor α and it may be attenuated by inflammation. Lipids Health Dis..

[31] Xu X., Fang H., Ji Q., Deng X., Dai T., Li J., Xiong D., Xie H., Liu X. (2025). Changes in alcohol consumption amoung Chinese adults from 2002 to 2015. J. Hyg. Res..

[32] Arranz S., Valderas-Martinez P., Chiva-Blanch G., Casas R., Urpi-Sarda M., Lamuela-Raventos R.M., Estruch R. (2013). Cardioprotective effects of cocoa: Clinical evidence from randomized clinical intervention trials in humans. Mol. Nutr. Food Res..

[33] Longnecker M.P., Tseng M. (1998). Alcohol, hormones, and postmenopausal women. Alcohol Health Res. World.

[34] Rimm E.B., Williams P., Fosher K., Criqui M., Stampfer M.J. (1999). Moderate alcohol intake and lower risk of coronary heart disease: Meta-analysis of effects on lipids and haemostatic factors. BMJ.

[35] Vatsalya V., Bin Liaquat H., Ghosh K., Mokshagundam S.P., McClain C.J. (2016). A Review on the Sex Differences in Organ and System Pathology with Alcohol Drinking. Curr. Drug Abus. Rev..

[36] Institute of Medicine, Committee on Health (2001). Health and Behavior: The Interplay of Biological, Behavioral, and Societal Influences.

[37] Erol A., Karpyak V.M. (2015). Sex and gender-related differences in alcohol use and its consequences: Contemporary knowledge and future research considerations. Drug Alcohol Depend..

[38] Huang S., Li J., Shearer G.C., Lichtenstein A.H., Zheng X., Wu Y., Jin C., Wu S., Gao X. (2017). Longitudinal study of alcohol consumption and HDL concentrations: A community-based study. Am. J. Clin. Nutr..

[39] Hannuksela M.L., Rämet M.E., Nissinen A.E., Liisanantti M.K., Savolainen M.J. (2004). Effects of ethanol on lipids and atherosclerosis. Pathophysiology.

[40] Kwon Y.-J., Kim S.E., Park B.-J., Bae J.-W., Kang H.-T. (2016). High-risk drinking is associated with dyslipidemia in a different way, based on the 2010–2012 KNHANES. Clin. Chim. Acta.

[41] De Oliveira e Silva E.R., Foster D., Harper M.M., Seidman C.E., Smith J.D., Breslow J.L., Brinton E.A. (2000). Alcohol consumption raises HDL cholesterol levels by increasing the transport rate of apolipoproteins A-I and A-II. Circulation.

[42] Beulens J.W.J., Sierksma A., van Tol A., Fournier N., van Gent T., Paul J.-L., Hendriks H.F.J. (2004). Moderate alcohol consumption increases cholesterol efflux mediated by ABCA1. J. Lipid Res..

[43] Muth N.D., Laughlin G.A., von Mühlen D., Smith S.C., Barrett-Connor E. (2010). High-density lipoprotein subclasses are a potential intermediary between alcohol intake and reduced risk of cardiovascular disease: The Rancho Bernardo Study. Br. J. Nutr..

[44] Kontush A., Chapman M.J. (2006). Antiatherogenic small, dense HDL—Guardian angel of the arterial wall?. Nat. Clin. Pract. Cardiovasc. Med..

[45] Cho K.-H., Nam H.-S., Kang D.-J., Park M.-H., Kim J.-H. (2022). Long-Term Alcohol Consumption Caused a Significant Decrease in Serum High-Density Lipoprotein (HDL)-Cholesterol and Apolipoprotein A-I with the Atherogenic Changes of HDL in Middle-Aged Korean Women. Int. J. Mol. Sci..

[46] Marmillot P., Munoz J., Patel S., Garige M., Rosse R.B., Lakshman M.R. (2007). Long-term ethanol consumption impairs reverse cholesterol transport function of high-density lipoproteins by depleting high-density lipoprotein sphingomyelin both in rats and in humans. Metabolism.

[47] Kechagias S., Zanjani S., Gjellan S., Leinhard O.D., Kihlberg J., Smedby Ö., Johansson L., Kullberg J., Ahlström H., Lindström T. (2011). Effects of moderate red wine consumption on liver fat and blood lipids: A prospective randomized study. Ann. Med..

[48] Chiva-Blanch G., Urpi-Sarda M., Ros E., Valderas-Martinez P., Casas R., Arranz S., Guillén M., Lamuela-Raventós R.M., Llorach R., Andres-Lacueva C. (2013). Effects of red wine polyphenols and alcohol on glucose metabolism and the lipid profile: A randomized clinical trial. Clin. Nutr..

